# Association Between Exercise Self-Efficacy and Health-Related Quality of Life Among Dialysis Patients: A Cross-Sectional Study

**DOI:** 10.3389/fpsyg.2022.875803

**Published:** 2022-06-20

**Authors:** Fan Zhang, Jing Liao, Weihong Zhang, Liuyan Huang

**Affiliations:** Department of Nephrology, Longhua Hospital, Shanghai University of Traditional Chinese Medicine, Shanghai, China

**Keywords:** exercise self-efficacy, physical activity, quality of life, dialysis, cross-sectional

## Abstract

**Background:**

Exercise self-efficacy is a vital determinant of an individual’s active participation in regular exercise, and exercise is a critical component of improving health-related quality of life (HRQOL) in dialysis patients. This study aimed to describe the relationship between exercise self-efficacy and HRQOL in dialysis patients.

**Materials and Methods:**

A cross-sectional study was conducted in Shanghai, China. Structured questionnaires distributed to the patients collected socio-demographic and disease-related information. Physical activity was assessed by a self-administered questionnaire, and the exercise self-efficacy scale (ESES) was used to measure exercise self-efficacy. HRQOL was evaluated by the kidney disease quality of life instrument-short form version 1.3 (KDQOL-SF™ v1.3). Data were analyzed using a univariate generalized linear model, Spearman correlation, and hierarchical multiple regression.

**Results:**

A positive association was observed between exercise self-efficacy and HRQOL (*r* = 0.310, *p* < 0.001). Physical activity as a predictor variable explained 9.8% of the variance in overall HRQOL (*p* < 0.001). Exercise self-efficacy explained an additional 7.1% of the HRQOL variance. In total, 24.6% of the variation in the HRQOL was explained by the socio-demographic variables, disease-related factors, physical activity, and exercise self-efficacy.

**Conclusion:**

Overall, only 16.9% of the change in HRQOL was explained by physical activity and exercise self-efficacy. Future research is still needed to further explore the factors influencing the HRQOL in dialysis patients. However, this finding suggests the need to consider the importance of HRQOL and physical activity as well as exercise self-efficacy when developing intervention programs.

## Introduction

Chronic kidney disease (CKD) is a progressive, debilitating illness defined by declining renal function that gradually progresses to end-stage renal disease (ESRD) (Webster et al., 2017). The prevalence of ESRD has increased over the past decade in parallel with an increasing prevalence of diabetes, hypertension, and aging (Rhee and Kovesdy, 2015; Cheng et al., 2021). Dialysis, including hemodialysis and peritoneal dialysis, is an effective renal replacement therapy modality for patients with ESRD. Over the next 5 years, the number of dialysis patients is estimated to be nearly 900,000 cases in China (Yang et al., 2021), and it has emerged as one of the most critical public health problems worldwide (Wang et al., 2019). Due to the complexity of the disease process and its management, dialysis treatment imposes significant security and financial burden on patients and their families, seriously affecting health-related quality of life (HRQOL) (Coresh, 2017).

Health-related quality of life is a primary patient-centered outcome and is increasingly used as an assessment metric for medical interventions (Segura-Orti et al., 2021). HRQOL can predict disease-related factors and assess prognosis, thus guiding the treatment of specific populations. Poor HRQOL was a common problem in dialysis patients and contributed to low survival rates and high emotional stress (Yapa et al., 2021). With this in mind, HRQOL is increasingly recognized as a key prognostic measure to evaluate the effectiveness of kidney disease treatment (Lightfoot et al., 2021).

Exercise self-efficacy, the confidence a person has in developing and meeting exercise goals, is vital to exercise motivation (O’Neil-Pirozzi, 2021). Results of a previous study showed that the level of exercise self-efficacy is low in patients with chronic diseases (Almutary and Tayyib, 2020), which was associated with poor HRQOL in some populations, e.g., chronic heart failure (Lee et al., 2017) and total knee arthroplasty recipients (Tobinaga et al., 2021). The literature indicated that exercise self-efficacy is a predictor of good HRQOL (Ogwumike et al., 2021), and low exercise self-efficacy often leads to physical inactivity, causing poor prognosis (Farris et al., 2016; Selzler et al., 2016; Kooijmans et al., 2020). In addition, good self-efficacy appears to be critical for improving HRQOL and health status, primarily when targeted secondary prevention strategies (Stevens et al., 2020).

Exercise self-efficacy is the confidence persons have in their ability to exercise and is an essential and modifiable predictor of physical activity and exercise behavior. The latter (exercise) are important non-pharmacological therapies to improve HRQOL in dialysis patients (Wilkinson et al., 2019). The association of exercise self-efficacy with HRQOL in dialysis patients remains unclear. To address and help bridge the gap between exercise self-efficacy and HRQOL, we conducted a cross-sectional study to explore the association between exercise self-efficacy and HRQOL in ESRD patients receiving dialysis treatment. This study was built based on the concepts of McAuley’s social cognitive theory (McAuley and Blissmer, 2000).

## Materials and Methods

### Study Design and Population

The study was conducted between October 2020 and June 2021. Using a convenience sampling method, samples were obtained from the nephrology department in Shanghai. The sample size was 5–10 times the number of scale entries. Considering a 10% inefficiency of the questionnaire, a minimum of 198 participants were required. The inclusion criteria were as follows: diagnosed with ESRD and already undergoing regular dialysis treatment; age older than 18 years; able to independently perform the activities of daily living (i.e., can walk at least 100 m alone without auxiliary equipment); able to understand and speak Chinese; consent to participate in the study. The exclusion criteria were cognitive impairment or mental disorders; refusal to participate. Patients who used a cane daily or had difficulty walking without a walking aid device were also excluded.

### Assessments

The assessment tools for collecting data included: (1) basic information questionnaire; (2) physical activity questionnaire; (3) exercise self-efficacy scale (ESES); (4) kidney disease quality of life (KDQOL) scale.

### Instruments

#### Basic Information Questionnaire

The socio-demographic questionnaire consisted of gender, age, marital status, education, and income. The disease-related factors included body mass index, causes for ESRD, dialysis modality, and dialysis vintage.

#### Physical Activity

Physical activity level was evaluated by reporting how often an individual exercised (≤ 1, 2–3, and ≥4 sessions per week) and the mean duration of each exercise session (<30, 30–45, 45–60, and >60 min per session). The physical activity (total minutes per week) was calculated according to the following formula: frequency (sessions per week) × duration (minutes per session) (Choi et al., 2019).

#### Exercise Self-Efficacy Scale

The ESES was used to measure exercise self-efficacy, developed by Bandura (Tung et al., 2005). For each item, individuals indicate their confidence to execute the behavior on a 100-point percentage scale comprised of 10-point increments, ranging from 0% (not at all confident) to 100% (highly confident). Total strength for each measure of self-efficacy is then calculated by summing the confidence ratings and dividing by the total number of items on the scale, resulting in a maximum possible efficacy score of 100. The Chinese version of the scale has good validity and reliability with a Cronbach’s alpha of 0.966 (Zhang and Xue, 2019).

#### Kidney Disease Quality of Life Scale

Kidney disease quality of life scale (KDQOL-SF™ v1.3), a 36-item disease-specific questionnaire, was employed to evaluate the patients’ HRQOL. The scale consists of five dimensions: physical component summary (PCS), mental component summary (MCS), burden of kidney disease, symptom/problem, and effects of kidney disease. The score ranged from 0 to 100, with higher numerical scores indicating better HRQOL. The Chinese version of the KDQOL-36™ has demonstrated acceptable levels of internal consistency (Cronbach’s α = 0.69–0.78) and test-retest reliability (ICC = 0.70–0.86) (Tao et al., 2014).

### Statistical Analysis

Categorical variables were presented as frequency (percentage) and continuous variables as mean ± standard deviations because the overall HRQOL score was normally distributed.

A univariate generalized linear model was used to analyze the relationship between each independent variable and HRQOL. Considering that the distribution of exercise self-efficacy scores was skewed, the Spearman correlation was used to assess the relationship between exercise self-efficacy and HRQOL.

In the hierarchical regression analysis, the model was entered in four blocks. Block 1 consisted of gender, age, marital status, education, and income, operationally defined as socio-demographic variables and entered first. Block 2 was disease-related factors, including body mass index, causes of ESRD, dialysis modality, and dialysis vintage. Block 3 included physical activity, and the exercise self-efficacy was added to block 4. After the entry of each block, the adjusted *R*^2^ change is observed to determine the proportion of variance described by HRQOL. The significant level was set at 0.05. Statistical analysis was performed using SPSS software (version 21.0).

## Results

### Basic Information

A total of 203 participants were finally included in this survey. Table 1 demonstrates the socio-demographic variables of the participants. The mean age was 58.57 ± 12.07 years, and more than half of the participants (*n* = 58.13%) had a lower physical activity (<1500 min/week).

**TABLE 1:** Characteristics of included patients according to socio-demographic variables (*n* = 203).

Variables	n (%)	β (95% CI)	*P*-value
**Gender**			
Male	114(56.16%)	–	
Female	89(43.84%)	−1.402 (−4.528, 1.729)	0.380
**Age (years)**			
<55	66(32.51%)	–	
≥55	137(67.49%)	0.023 (−0.108, 0.153)	0.731
**Marital status**			
Non-married	21(10.34%)	–	
Married	162(79.80%)	4.713 (−0.378, 9.805)	0.070
Widowed	20(9.85%)	5.395 (−1.464, 12.255)	0.123
**Educational**			
Primary school or below	18(8.87%)	–	
Junior high school	86(42.36%)	−1.314 (−7.041, 4.412)	0.362
Senior high school	65(32.02%)	−1.520 (−7.404, 4.365)	0.613
Undergraduate or above	34(16.75%)	−2.996 (−9.436, 3.444)	0.653
**Income**			
Poor	50(24.63%)	–	
Moderate	66(32.51%)	3.900 (−0.200, 7.999)	0.062
Good	56(27.59%)	0.236 (−4.019, 4.490)	0.913
Very good	31(15.27%)	0.034 (−4.965, 5.033)	0.989
**Causes**			
Glomerulonephritis	77(37.93%)	–	
Diabetic nephropathy	47(23.15%)	0.032 (−4.057, 4.121)	0.988
Hypertensive nephropathy	35(17.24%)	0.821 (−3.683, 5.325)	0.721
IgA nephropathy	22(10.84%)	1.945 (−3.396, 7.285)	0.475
Other	22(10.84%)	1.855 (−3.486, 7.196)	0.496
**Body mass index**			
Underweight	14(6.90%)	–	
Normal	115(56.65%)	2.711 (−3.525, 8.946)	0.394
Overweight	62(30.54%)	2.034 (−4.484, 8.552)	0.541
Obesity	12(5.91%)	−1.617 (−10.283, 7.048)	0.715
**Dialysis modality**			
Peritoneal dialysis	116(58.13%)	–	
Hemodialysis	87(42.86%)	4.443 (1.363, 7.523)	0.005
**Dialysis vintage (months)**			
<36	78(38.42%)	–	
36–60	69(33.99%)	−3.780 (−7.403, −0.158)	0.041
>60	56(27.59%)	−1.546 (−5.385, 2.293)	0.430
**Physical activity (min/week)**			
<1500	118(58.13%)	–	
≥1500	85(41.87%)	3.842 (0.706, 6.978)	0.016

### Health-Related Quality of Life

**FIGURE 1:**
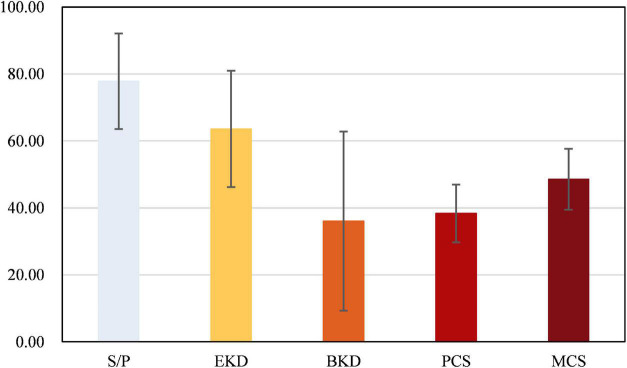
Scores on the five dimensions of the kidney disease quality of life (KDQOL) scale. SP, symptom/problem; EKD, effects of kidney disease; BKD, burden of kidney disease; PCS, physical component summary; MCS, mental component summary.

**FIGURE 2:**
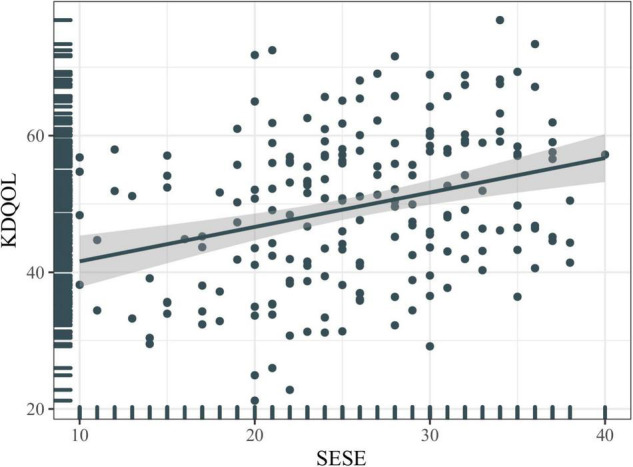
Correlation between exercise self-efficacy and health-related quality of life (HRQOL). KDQOL, kidney disease quality of life; SESE, exercise self-efficacy scale.

The mean score of dialysis patients’ HRQOL was 49.51 ± 11.33. The comparison of the mean scores for each dimension of the scale in the study population showed that “burden of kidney disease” (38.08 ± 26.73) had the lowest mean score, and “symptom/problem” (77.85 ± 14.28) had the highest mean score among all the studied dimensions (Figure 1). Figure 2 shows the Spearman correlation between exercise self-efficacy and HRQOL, and a positive association was observed (*r* = 0.310, *p* < 0.001).

### Hierarchical Regression Models

The results of the hierarchical regression models are shown in Table 2. In model 1, socio-demographic could explain 2.3% of the variation in KDQOL. For model 2, disease-related factors have an explanatory strength of 5.3% for KDQOL, and the *R*^2^ increased from 0.023 to 0.076. Model 3 showed a significant change in *F*-value (*p* < 0.05) with the addition of physical activity to model 2, implying that physical activity has an explanatory significance to the model. Moreover, the *R*^2^ value increased from 0.076 to 0.174, implying that physical activity can generate an explanatory strength of 9.8% for KDQOL. Specifically, the regression coefficient value for physical activity was 8.242 (*t* = 4.772, *p* < 0.001), implying that physical activity can significantly positively correlate with KDQOL. In model 4, exercise self-efficacy was added, which explained an additional 7.1% variation, with the *R*^2^ value increasing to 0.246. The regression coefficient value of exercise self-efficacy was 0.472 (*t* = 4.234, *p* < 0.001), implying that exercise self-efficacy could significantly correlate with KDQOL.

**TABLE 2:** Hierarchical linear regression for prediction of health-related quality of life (HRQOL).

	Model 1	Model 2	Model 3	Model 4
Sex	−1.641(−0.995)	−1.418(−0.859)	−1.094(−0.699)	−0.939(−0.626)
Age	1.276 (0.682)	0.418 (0.221)	2.526 (1.366)	2.834 (1.599)
Income	−0.413(−0.490)	1.140 (1.193)	1.399 (1.542)	1.209 (1.388)
Education	−0.589(−0.574)	−1.736(−1.609)	−1.877(−1.834)	−1.277(−1.289)
Marry	2.205 (1.163)	2.189 (1.165)	3.955*(2.174)	3.074 (1.751)
Dialysis		5.515**(2.980)	7.614**(4.209)	7.912**(4.561)
Dialysis vintage		−0.699(−0.708)	−1.159(−1.232)	−1.423(−1.575)
Morbidity		0.479 (0.819)	0.751 (1.348)	0.555 (1.035)
Body mass index		−1.005(−0.860)	−1.742(−1.557)	−1.766(−1.648)
Physical activity			8.242**(4.772)	6.703**(3.957)
SESE				0.472**(4.254)
*R* ^2^	0.023	0.076	0.174	0.246
Adjusted *R*^2^	–0.002	0.033	0.131	0.202
*F*	*F* (5,197) = 0.924	*F* (9,193) = 1.773	*F* (10,192) = 4.053	*F* (11,191) = 5.657
*P*-value for *F*	*P* = 0.466	*P* = 0.076	*P* < 0.001	*P* < 0.001
Δ*R*^2^	0.023	0.053	0.098	0.071
Δ*F*	*F* (5,197) = 0.924	*F* (4,193) = 2.791	*F* (1,192) = 22.773	*F* (1,191) = 18.096

*p < 0.05, **p < 0.001. Values in the parenthesis are t values. R^2^ indicates the explanatory power of the model. The F-value was used to determine if the fitting performance of the model was statistically acceptable.

## Discussion

As dialysis technology is improving and kidney disease patients are living longer, healthcare providers are placing greater emphasis on patients’ HRQOL.

A recent meta-analysis involving 147 studies reported a pooling mean HRQOL score for patients undergoing dialysis as 64.25 [95% confidence interval (CI): 55.67–72.82] (Raoofi et al., 2021). In the present study, the overall mean score of HRQOL was modestly lower than the results mentioned above. The reason could be that the patients enrolled in this study were older with an average age of 58.57 ± 12.07 years. As described in the study by Ishiwatari et al. (2020), dialysis patients’ HRQOL decreased over time, especially among those older adults. In the aging process, frailty predisposes to develop in elderly dialysis patients, increasing the risk of adverse events such as functional impairment, limited ability to perform activities of daily living, and falling, causing a decrease in HRQOL (Li et al., 2021; Guo et al., 2022).

Compared to a large sample report from the United States, the results of this study showed lower scores for all dimensions except MCS (Cohen et al., 2019). This may be related to the faster development of dialysis technology in the United States (Kurella Tamura et al., 2018). In addition, the results of low scores in each dimension may be related to the kidney disease itself and existing comorbidities and related disease complications. The lowest burden of kidney disease score was similar to the Peritoneal and Dialysis Outcomes and Practice Patterns Studies (Brown et al., 2021), indicating that disease burden is an important cause of lower HRQOL for dialysis patients.

As concluded by Abeywickrama et al. (2020), symptom burden scores independently affect HRQOL (PCS: β = 0.417; MCS: β = 0.464). In the univariate analysis, dialysis type, dialysis vintage, and physical activity represented predictors for HRQOL in dialysis patients. Unlike previous studies (Chuasuwan et al., 2020), the results of this study showed that the HRQOL scores of hemodialysis patients were 4.443 [odds ratio (OR): 1.363, 7.523; *p* = 0.005] higher than those of peritoneal dialysis patients, but similar to the results of de Abreu et al. (2011) and Gonçalves et al. (2015). This happened because of the limited sample size and the different inclusion criteria.

Regarding dialysis vintage, we found that patients with longer dialysis vintage had lower HRQOL scores than those with shorter ones. Early studies have shown that HRQOL decreases after dialysis initiation in patients with kidney disease (Eneanya et al., 2019; Jung et al., 2019). The number of medications and co-morbidities increases with dialysis vintage, further eroding HRQOL (Al-Mansouri et al., 2021). Therefore, Boini et al. (2011) emphasized that HRQOL at dialysis initiation is significantly influenced by the quality of predialysis nephrology care and that emphasis should be placed on disease management.

The relationship of interest between physical activity and the prognosis of dialysis patients has been widely recognized (Zelle et al., 2017). As in previous studies, this study showed that patients who participated in higher physical activity levels had higher HRQOL scores than those who were less physically active than recommended. Previous studies have shown that physical activity increases the HRQOL of dialysis patients by improving cardiovascular health, inflammatory status, physical fitness, and reducing disease-related symptoms (Wilkinson et al., 2016; Manfredini et al., 2017; Sheshadri et al., 2019).

Moreover, a significant positive interrelation was found in the current study between exercise self-efficacy and HRQOL. This positive relationship corroborates the social cognitive model proposed by McAuley and Blissmer (2000) that exercising self-efficacy has a positive effect on PCS and MCS. Self-efficacy has been reported to be the most dominant factor in the uptake and maintenance of exercise in populations with chronic conditions (Rajati et al., 2014; Selzler et al., 2020b). Furthermore, self-efficacy is an essential determinant of health behavior, associated with a positive HRQOL among patients living with chronic illnesses (Peters et al., 2019; Selzler et al., 2020a).

In the stratified regression analysis, our study presented that physical activity remained a statistically significant positive correlation with HRQOL. Also, physical activity as a predictor variable can explain an additional 9.8% of the variation in HRQOL. Most of the studies found a positive relationship between physical activity and HRQOL as well as a negative relationship between sedentary behavior and HRQOL among dialysis patients (Kang et al., 2017; Tsai et al., 2017; Hornik and Duława, 2019). The present study further confirmed this positive finding. Moreover, we found that exercise self-efficacy was an important predictor of HRQOL in dialysis patients. Exercise self-efficacy is a strong determinant of behavior in physical activity and other health domains and has been shown to correlate with HRQOL in other populations (Selzler et al., 2016; Klompstra et al., 2018; Stevens et al., 2020). This study reinforces existing evidence on the importance of self-efficacy as a determinant of HRQOL in patients with chronic disease.

Frailty is a state of increased susceptibility to physical stressors and is highly prevalent in dialysis patients (Zhao et al., 2020). Frailty leads to limited physical capacity and low adherence to exercise, i.e., low exercise self-efficacy (Nitta et al., 2018). It has been confirmed that exercise-based physical activity is a major strategy to combat frailty in dialysis patients, improving physical fitness, muscle mass, and strength and regulating physiological condition (Wilkinson et al., 2020; Nixon et al., 2021), increased practitioner movement self-efficacy, and increased physical self-efficacy, which leads to higher exercise self-efficacy. Therefore, interventions aiming at increasing the HRQOL of dialysis patients should consider physical activity and exercise self-efficacy in these populations.

Our study has several limitations. Firstly, conducted in a single center, the results limit generalizability. This study focused only on a sample of the dialysis population and has limited universality to non-dialysis CKD patients. Secondly, self-administered questionnaires were used to assess physical activity; an inaccurate estimation and recall bias was unavoidable. Thirdly, this study used a cross-sectional design and could not infer a causal relationship between exercise self-efficacy and HRQOL. Fourthly, the convenience sampling method could bring the disadvantages of possible selection and researcher bias. Finally, we were unable to control the intensity of physical activity, and since we did not collect relevant data, this may have confounded the relationship between key variables.

## Conclusion

The present study revealed that overall HRQOL in dialysis patients was low, while the burden of kidney disease dimension was the most impacted. Dialysis modality, dialysis vintage, physical activity, and exercise self-efficacy were significantly associated with poor overall HRQOL in our study population. In total, only 16.9% of the variation of HRQOL was explained by physical activity and exercise self-efficacy, and future studies still need to expand the sample size to explore the potential predictors further. Nonetheless, this finding recommends considering HRQOL and physical activity and exercise self-efficacy when developing intervention programs.

## Data Availability Statement

The raw data supporting the conclusions of this article will be made available by the authors, without undue reservation.

## Ethics Statement

The studies involving human participants were reviewed and approved by Longhua Hospital Shanghai University of Traditional Chinese Medicine. The patients/participants provided their written informed consent to participate in this study. Written informed consent was obtained from the individual(s) for the publication of any potentially identifiable images or data included in this article.

## Author Contributions

FZ and JL worked on the conception and designed of the study. WZ, JL, and LH worked on data collection. FZ worked on the data analysis and wrote the first draft. LH revised the draft. All the authors have approved the final version for publication.

## Conflict of Interest

The authors declare that the research was conducted in the absence of any commercial or financial relationships that could be construed as a potential conflict of interest.

## Publisher’s Note

All claims expressed in this article are solely those of the authors and do not necessarily represent those of their affiliated organizations, or those of the publisher, the editors and the reviewers. Any product that may be evaluated in this article, or claim that may be made by its manufacturer, is not guaranteed or endorsed by the publisher.

## References

[B1] Abeywickrama H. M. Wimalasiri S. Koyama Y. Uchiyama M. Shimizu U. Kakihara N. (2020). Quality of life and symptom burden among chronic kidney disease of uncertain etiology (CKDu) patients in Girandurukotte, Sri Lanka. *Int. J. Environ. Res. Public Health* 17:4041. 10.3390/ijerph17114041 32517110 PMC7312904

[B2] Al-Mansouri A. Al-Ali F. S. Hamad A. I. Mohamed Ibrahim M. I. Kheir N. Ibrahim R. A. (2021). Assessment of treatment burden and its impact on quality of life in dialysis-dependent and pre-dialysis chronic kidney disease patients in Qatar. *Res. Social Adm. Pharm*. 17 1937–1944. 10.1016/j.sapharm.2021.02.010 33612446

[B3] Almutary H. Tayyib N. (2020). Factors associated with exercise self-efficacy among people with chronic diseases. *Appl. Nurs. Res.* 54:151275. 10.1016/j.apnr.2020.151275 32650891

[B4] Boini S. Frimat L. Kessler M. Briançon S. Thilly N. (2011). Predialysis therapeutic care and health-related quality of life at dialysis onset (The pharmacoepidemiologic AVENIR study). *Health Qual. Life Outcomes* 9:7. 10.1186/1477-7525-9-7 21261936 PMC3036597

[B5] Brown E. A. Zhao J. Mccullough K. Fuller D. S. Figueiredo A. E. Bieber B. (2021). Burden of kidney disease, health-related quality of life, and employment among patients receiving peritoneal dialysis and in-center hemodialysis: findings from the DOPPS program. *Am. J. Kidney Dis*. 78 489–500.e1. 10.1053/j.ajkd.2021.02.327 33872688

[B6] Cheng H. T. Xu X. Lim P. S. Hung K. Y. (2021). Worldwide epidemiology of diabetes-related end-stage renal disease, 2000-2015. *Diabetes Care* 44 89–97. 10.2337/dc20-1913 33203706

[B7] Choi J. Choi J. Y. Lee S. A. Lee K. M. Shin A. Oh J. (2019). Association between family history of diabetes and clusters of adherence to healthy behaviors: cross-sectional results from the Health Examinees-Gem (HEXA-G) study. *BMJ Open* 9:e025477. 10.1136/bmjopen-2018-025477 31209083 PMC6588964

[B8] Chuasuwan A. Pooripussarakul S. Thakkinstian A. Ingsathit A. Pattanaprateep O. (2020). Comparisons of quality of life between patients underwent peritoneal dialysis and hemodialysis: a systematic review and meta-analysis. *Health Qual. Life Outcomes* 18:191. 10.1186/s12955-020-01449-2 32552800 PMC7302145

[B9] Cohen D. E. Lee A. Sibbel S. Benner D. Brunelli S. M. Tentori F. (2019). Use of the KDQOL-36™ for assessment of health-related quality of life among dialysis patients in the United States. *BMC Nephrol.* 20:112. 10.1186/s12882-019-1295-0 30935377 PMC6444438

[B10] Coresh J. (2017). Update on the burden of CKD. *J. Am. Soc. Nephrol.* 28 1020–1022. 10.1681/asn.2016121374 28302756 PMC5373470

[B11] de Abreu M. M. Walker D. R. Sesso R. C. Ferraz M. B. (2011). Health-related quality of life of patients recieving hemodialysis and peritoneal dialysis in São Paulo, Brazil: a longitudinal study. *Value Health* 14 S119–S121. 10.1016/j.jval.2011.05.016 21839882

[B12] Eneanya N. D. Maddux D. W. Reviriego-Mendoza M. M. Larkin J. W. Usvyat L. A. Van Der Sande F. M. (2019). Longitudinal patterns of health-related quality of life and dialysis modality: a national cohort study. *BMC Nephrol.* 20:7. 10.1186/s12882-018-1198-5 30621634 PMC6325821

[B13] Farris S. G. Davis M. L. Rosenfield D. Kauffman B. Y. Baird S. O. Powers M. B. (2016). Exercise self-efficacy moderates the relation between anxiety sensitivity and body mass index and exercise tolerance in treatment-seeking smokers. *Ment. Health Phys. Act.* 10 25–32. 10.1016/j.mhpa.2016.05.001 27725844 PMC5055124

[B14] Gonçalves F. A. Dalosso I. F. Borba J. M. Bucaneve J. Valerio N. M. Okamoto C. T. (2015). Quality of life in chronic renal patients on hemodialysis or peritoneal dialysis: a comparative study in a referral service of Curitiba - PR. *J. Bras. Nefrol.* 37 467–474. 10.5935/0101-2800.20150074 26648496

[B15] Guo Y. Tian R. Ye P. Luo Y. (2022). Frailty in older patients undergoing hemodialysis and its association with all-cause mortality: a prospective cohort study. *Clin. Interv. Aging* 17 265–275. 10.2147/cia.S357582 35313671 PMC8934156

[B16] Hornik B. Duława J. (2019). Frailty, quality of life, anxiety, and other factors affecting adherence to physical activity recommendations by hemodialysis patients. *Int. J. Environ. Res. Public Health* 16:1827. 10.3390/ijerph16101827 31126041 PMC6571908

[B17] Ishiwatari A. Yamamoto S. Fukuma S. Hasegawa T. Wakai S. Nangaku M. (2020). Changes in quality of life in older hemodialysis patients: a cohort study on dialysis outcomes and practice patterns. *Am. J. Nephrol.* 51 650–658. 10.1159/000509309 32739911 PMC7592938

[B18] Jung H. Y. Jeon Y. Park Y. Kim Y. S. Kang S. W. Yang C. W. (2019). Better quality of life of peritoneal dialysis compared to hemodialysis over a two-year period after dialysis initiation. *Sci. Rep.* 9:10266. 10.1038/s41598-019-46744-1 31312004 PMC6635359

[B19] Kang S. H. Do J. Y. Jeong H. Y. Lee S. Y. Kim J. C. (2017). The clinical significance of physical activity in maintenance dialysis patients. *Kidney Blood Press. Res.* 42 575–586. 10.1159/000480674 29017151

[B20] Klompstra L. Jaarsma T. Strömberg A. (2018). Self-efficacy mediates the relationship between motivation and physical activity in patients with heart failure. *J. Cardiovasc. Nurs.* 33 211–216. 10.1097/jcn.0000000000000456 29189427 PMC5908261

[B21] Kooijmans H. Post M. Motazedi E. Spijkerman D. Bongers-Janssen H. Stam H. (2020). Exercise self-efficacy is weakly related to engagement in physical activity in persons with long-standing spinal cord injury. *Disabil. Rehabil.* 42 2903–2909. 10.1080/09638288.2019.1574914 30907149

[B22] Kurella Tamura M. Thomas I. C. Montez-Rath M. E. Kapphahn K. Desai M. Gale R. C. (2018). Dialysis initiation and mortality among older veterans with kidney failure treated in medicare vs the department of veterans affairs. *JAMA Intern. Med.* 178 657–664. 10.1001/jamainternmed.2018.0411 29630695 PMC6583073

[B23] Lee H. Boo S. Yu J. Suh S. R. Chun K. J. Kim J. H. (2017). Physical functioning, physical activity, exercise self-efficacy, and quality of life among individuals with chronic heart failure in korea: a cross-sectional descriptive study. *J. Nurs. Res.* 25 131–139. 10.1097/jnr.0000000000000150 28277393

[B24] Li Y. Zhang D. Ma Q. Diao Z. Liu S. Shi X. (2021). The impact of frailty on prognosis in elderly hemodialysis patients: a prospective cohort study. *Clin. Interv. Aging* 16 1659–1667. 10.2147/cia.S329665 34552324 PMC8450604

[B25] Lightfoot C. J. Howell M. Smith A. C. (2021). How to assess quality of life in persons with chronic kidney disease. *Curr. Opin. Nephrol. Hypertens*. 30 547–554. 10.1097/mnh.0000000000000740 34433189

[B26] Manfredini F. Mallamaci F. D’arrigo G. Baggetta R. Bolignano D. Torino C. (2017). Exercise in patients on dialysis: a multicenter, randomized clinical trial. *J. Am. Soc. Nephrol.* 28 1259–1268. 10.1681/asn.2016030378 27909047 PMC5373448

[B27] McAuley E. Blissmer B. (2000). Self-efficacy determinants and consequences of physical activity. *Exerc. Sport Sci. Rev.* 28 85–88. 10902091

[B28] Nitta K. Hanafusa N. Tsuchiya K. (2018). Role of frailty on outcomes of dialysis patients. *Contrib. Nephrol.* 195 102–109. 10.1159/000486940 29734155

[B29] Nixon A. C. Bampouras T. M. Gooch H. J. Young H. M. L. Finlayson K. W. Pendleton N. (2021). Home-based exercise for people living with frailty and chronic kidney disease: a mixed-methods pilot randomised controlled trial. *PLoS One* 16:e0251652. 10.1371/journal.pone.0251652 34197486 PMC8248609

[B30] O’Neil-Pirozzi T. M. (2021). Cognitive exercise self-efficacy of community-dwelling older adults: measurement and associations with other self-reported cognitive exercise factors. *Brain Sci.* 11:672. 10.3390/brainsci11060672 34063967 PMC8224066

[B31] Ogwumike O. O. Omoregie A. A. Dada O. O. Badaru U. M. (2021). Quality of life of stroke survivors: a cross-sectional study of association with functional independence, self-reported fatigue and exercise self-efficacy. *Chronic Illness.* 10.1177/17423953211023960 [Epub ahead of print]. 34120490

[B32] Peters M. Potter C. M. Kelly L. Fitzpatrick R. (2019). Self-efficacy and health-related quality of life: a cross-sectional study of primary care patients with multi-morbidity. *Health Qual. Life Outcomes* 17:37. 10.1186/s12955-019-1103-3 30764833 PMC6376655

[B33] Rajati F. Sadeghi M. Feizi A. Sharifirad G. Hasandokht T. Mostafavi F. (2014). Self-efficacy strategies to improve exercise in patients with heart failure: a systematic review. *ARYA Atheroscler.* 10 319–333. 25815022 PMC4354085

[B34] Raoofi S. Pashazadeh Kan F. Rafiei S. Hoseinipalangi Z. Rezaei S. Ahmadi S. (2021). Hemodialysis and peritoneal dialysis-health-related quality of life: systematic review plus meta-analysis. *BMJ Support. Palliat. Care* bmjspcare-2021-003182. 10.1136/bmjspcare-2021-003182 [Epub ahead of print]. 34301643

[B35] Rhee C. M. Kovesdy C. P. (2015). Epidemiology: spotlight on CKD deaths—increasing mortality worldwide. *Nat. Rev. Nephrol.* 11 199–200. 10.1038/nrneph.2015.25 25734769 PMC4379111

[B36] Segura-Orti E. Koufaki P. Kouidi E. (2021). Bridging the gap from research to practice for enhanced health-related quality of life in people with chronic kidney disease. *Clin. Kidney J.* 14 ii34–ii42. 10.1093/ckj/sfaa268 33981418 PMC8101625

[B37] Selzler A. M. Rodgers W. M. Berry T. R. Stickland M. K. (2020b). Coping versus mastery modeling intervention to enhance self-efficacy for exercise in patients with COPD. *Behav. Med.* 46 63–74. 10.1080/08964289.2018.1561411 30758267

[B38] Selzler A. M. Habash R. Robson L. Lenton E. Goldstein R. Brooks D. (2020a). Self-efficacy and health-related quality of life in chronic obstructive pulmonary disease: a meta-analysis. *Patient Educ. Couns.* 103 682–692. 10.1016/j.pec.2019.12.003 31859120

[B39] Selzler A. M. Rodgers W. M. Berry T. R. Stickland M. K. (2016). The importance of exercise self-efficacy for clinical outcomes in pulmonary rehabilitation. *Rehabil. Psychol.* 61 380–388. 10.1037/rep0000106 27831730

[B40] Sheshadri A. Kittiskulnam P. Johansen K. L. (2019). Higher physical activity is associated with less fatigue and insomnia among patients on hemodialysis. *Kidney Int. Rep.* 4 285–292. 10.1016/j.ekir.2018.10.014 30775625 PMC6365400

[B41] Stevens A. Stanton R. Rebar A. L. (2020). Helping people with parkinson disease build exercise self-efficacy. *Phys. Ther.* 100 205–208. 10.1093/ptj/pzz160 31665447

[B42] Tao X. Chow S. K. Wong F. K. (2014). Determining the validity and reliability of the Chinese version of the kidney disease quality of life questionnaire (KDQOL-36™). *BMC Nephrol.* 15:115. 10.1186/1471-2369-15-115 25015224 PMC4115482

[B43] Tobinaga T. Obayashi S. Miyazaki C. Yazawa M. Saito T. Hashimoto K. (2021). The impact of self-efficacy for physical activity on health-related quality of life in total knee arthroplasty recipients. *J. Back Musculoskelet. Rehabil*. 34 829–835. 10.3233/bmr-200017 33935059

[B44] Tsai Y. C. Chen H. M. Hsiao S. M. Chen C. S. Lin M. Y. Chiu Y. W. (2017). Association of physical activity with cardiovascular and renal outcomes and quality of life in chronic kidney disease. *PLoS One* 12:e0183642. 10.1371/journal.pone.0183642 28832653 PMC5568323

[B45] Tung W. C. Gillett P. A. Pattillo R. E. (2005). Applying the Transtheoretical Model to physical activity in family caregivers in Taiwan. *Public Health Nurs.* 22 299–310. 10.1111/j.0737-1209.2005.220405.x 16150011

[B46] Wang F. Yang C. Long J. Zhao X. Tang W. Zhang D. (2019). Executive summary for the 2015 annual data report of the china kidney disease network (CK-NET). *Kidney Int.* 95 501–505. 10.1016/j.kint.2018.11.011 30784660

[B47] Webster A. C. Nagler E. V. Morton R. L. Masson P. (2017). Chronic kidney disease. *Lancet* 389 1238–1252. 10.1016/s0140-6736(16)32064-5 27887750

[B48] Wilkinson T. J. Mcadams-Demarco M. Bennett P. N. Wilund K. (2020). Advances in exercise therapy in predialysis chronic kidney disease, hemodialysis, peritoneal dialysis, and kidney transplantation. *Curr. Opin. Nephrol. Hypertens.* 29 471–479. 10.1097/mnh.0000000000000627 32701595 PMC7526394

[B49] Wilkinson T. J. Shur N. F. Smith A. C. (2016). “Exercise as medicine” in chronic kidney disease. *Scand. J. Med. Sci. Sports* 26 985–988. 10.1111/sms.12714 27334146

[B50] Wilkinson T. J. Watson E. L. Gould D. W. Xenophontos S. Clarke A. L. Vogt B. P. (2019). Twelve weeks of supervised exercise improves self-reported symptom burden and fatigue in chronic kidney disease: a secondary analysis of the ‘ExTra CKD’ trial. *Clin. Kidney J.* 12 113–121. 10.1093/ckj/sfy071 30746138 PMC6366144

[B51] Yang C. Yang Z. Wang J. Wang H. Y. Su Z. Chen R. (2021). Estimation of prevalence of kidney disease treated with dialysis in China: a study of insurance claims data. *Am. J. Kidney Dis.* 77 889–897.e1. 10.1053/j.ajkd.2020.11.021 33421457

[B52] Yapa H. E. Purtell L. Chambers S. Bonner A. (2021). Alterations in symptoms and health-related quality of life as kidney function deteriorates: a cross-sectional study. *J. Clin. Nurs.* 30 1787–1796. 10.1111/jocn.15738 33656217

[B53] Zelle D. M. Klaassen G. Van Adrichem E. Bakker S. J. Corpeleijn E. Navis G. (2017). Physical inactivity: a risk factor and target for intervention in renal care. *Nat. Rev. Nephrol.* 13 152–168. 10.1038/nrneph.2016.187 28138130

[B54] Zhang Y. Xue G. (2019). [Reliability and validity test of Exercise Self-Efficacy Scale in maintenance hemodialysis patients]. *Chin. Nurs. Res.* 33 3133–3136.

[B55] Zhao Y. Liu Q. Ji J. (2020). The prevalence of frailty in patients on hemodialysis: a systematic review and meta-analysis. *Int. Urol. Nephrol.* 52 115–120. 10.1007/s11255-019-02310-2 31642001

